# Rheological Characterization and Accumulation Tests for Strong Thixotropic Engineering Slurry

**DOI:** 10.3390/ma15196891

**Published:** 2022-10-04

**Authors:** Kekuo Yuan, Yating Lu, Wanlu Li, Hongdan Yu, Shan Gao

**Affiliations:** 1 College of Civil Engineering, Xijing University, Xi’an 710123, China; 2 Shanxi Key Laboratory of Safety and Durability of Concrete Structures, Xi’an 710123, China; 3 State Key Laboratory of Geomechanics and Geotechnical Engineering, Wuhan 430071, China; 4 School of Civil Engineering, Harbin Institute of Technology, Harbin 150090, China

**Keywords:** rheological engineering slurry, thixotropic characterization, rheological model, accumulation model

## Abstract

Underground void subsidence hazards, especially mine goaf, have now become one of the major social problems affecting the well-being of civilians and development in China. The objective of this study was to propose a kind of strong thixotropic engineering slurry and filling treatment for use in underground void subsidence hazards. The optimal agent ratio for thixotropic cement slurry/mortar was obtained by indoor tests, where the rheological agent is 7.5% compared to the weight of cement, the water–solid ratio is in the range of 0.7~0.8, and the aeolian sand ratio can be 0~1.5 times that of cement. The rheological properties of slurry and mortar were tested using a Brookfield RST-SST rheometer. The results show that aeolian sand can be used as thixotropic cement mortar for a backfilling treatment for underground voids (mine goaf). The static yield stress increases non-linearly compared to existing thixotropic models. The stress decays sharply with shearing (the shear rate is more or less 10 s^−1^) and then the stress increases with the increase in shear rate (the shear rate is more than 10 s^−1^). The increase in the stress of the slurry is greater than in the mortar. A natural logarithmic function between yield stress and rest time (only 1 parameter), an exponential function with two parts for stress–shear rate (a rheology model, with only 3 parameters), and an exponential function for the accumulation law (only 2 parameters) were proposed in turn.

## 1. Introduction

China is rich in coal seams, with 150 coal-mining regions and over 426 mining cities. Underground void subsidence disasters have occurred frequently in China [1]; these are triggered by coal mining [2]. It can be envisioned that a large amount of farmland and infrastructures will be constructed or operated in the mined areas. The traditional strategies of moving operations from the mined areas are anachronistic and inappropriate [3]. Stability control of the rock above the mined-out or karst cavities is one of the most challenging problems, while backfilling is the most reliable and effective treatment scheme [4].

However, in the process of backfilling treatments for these underground voids, the filling materials are in great demand and are highly costly over tens of thousands of cubic meters. Filling treatment materials, such as high-content fly ash cement slurry and cement clay slurry, have been developed [5], while a backfilling method with intermittent grouting and the addition of sodium silicate was adopted [6]. A form of super-high-water material was studied and put forward in four kinds of filling and mining technologies, namely, the open type, bag type, mixed type, and segmented barrier type [7]. However, there are some deficiencies in the materials used for goaf filling treatments at present. For solid fillings, the compactness of the solid accumulation is difficult to achieve [8]. Traditional conventional slurry, which is made of cement and fly ash, has several shortcomings, such as too great a liquidity, a high bleeding rate, low concretion rate, large water dispersion, high costs, and uncontrollable effects; all these drawbacks seriously restrict the refinement and high efficiency of goaf treatment engineering [9]. In the case of chemical slurry, which is toxic and of high cost, the equipment and operation processes are complex and so it is difficult to use this to fill large-cavity areas. With the paste-like materials, it is difficult to meet the requirements of goaf cavity filling because of their low liquidity and high demand for delivery devices [10].

**Figure 1:**
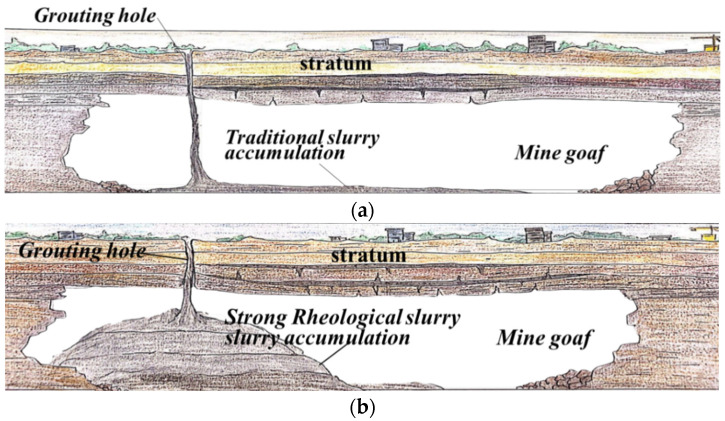
Schematic diagram of slurry accumulation by grouting and filling in the goaf: (**a**) traditional slurry; (**b**) strong rheological engineering slurry.

The ideal slurry for mine-out area backfilling should have high fluidity in the process of mixing and pumping [11] and should quickly lose its fluidity and present a solid-like state when the flow velocity is very low or static after being pumped into the underground cavities; there should be no agglomeration or segregation, even it is static for a long time. If it can flow in a very limited range into large, empty cavities and pile up at a steep angle, this means that it can support a finite amount of stress without flowing. However, the macroscopic flow is achieved as soon as the stress applied to the system overcomes that which can be supported by the network of particles in interaction. This critical value is called yield stress and is the dominant intrinsic parameter of what is called, in practice, the workability of slurry or concrete. These criteria validate the practical value and great application prospects in the field of backfill treatment of mined-out cavities (Figure 1). This kind of physical property is called thixotropy, which is a rheological phenomenon wherein the viscosity or shear force of the slurry changes with time and shear rate. The time-dependent behaviors of yield stress can contribute to the build-up of yield stress and result in the formation of semi-stagnant, semi-solidified slurry.

When the hydration process is neglected, thixotropic cement slurry can be regarded as a general thixotropic fluid [12]. The rapid build-up and the law of yield stress when they are at rest, along with the stress decay law when they are sheared [13], are the main properties of thixotropic slurries.

A time-dependent expression for the thixotropic areas was proposed from the study of the thixotropic behavior of Aerosil 200 hydrogels at different concentrations [14], but the development of yield stress in the slurries as the rest time increased had not been established and the solids concentration was not considered. The behavior of suspensions of coarse particles in a thixotropic cement paste was studied to relate the yield stress of these mixtures to the yield stress of the suspending cement paste, to the time passed at rest, and to the coarse particle volume fraction. A conclusion was given that the yield stress of the suspension does not depend on the physicochemical properties of the suspending yield stress fluid; it only depends on its yield stress value [15]. The thixotropy and structural breakdown of self-consolidating concrete (SCC) mixtures containing various supplementary cementitious materials (SCM) were investigated using different approaches [16]. In terms of the thixotropic behavior of standard fresh cement pastes, some studies [17] showed that a short-term thixotropy exists due to colloidal flocculation, along with long-term thixotropy (which is of practical interest) due to the ongoing hydrate nucleation.

**Figure 2:**
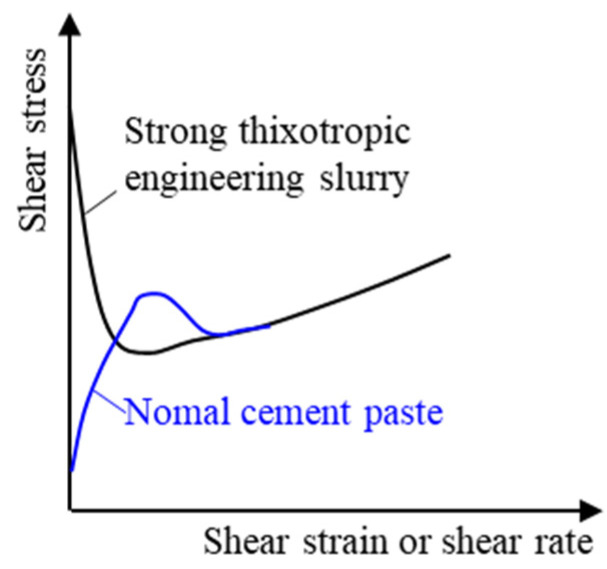
Different shear stress–shear strain curves.

However, a strong difference exists in the stress-shear strain curves of cement pastes [11,18] and strong thixotropic engineering slurry (Figure 2); the shear stress decreases from yield stress instantaneously, with little scale shear strain in the strong thixotropic slurry.

The aim of the first part of this paper is to develop a new type of thixotropic engineering slurry, with recognized economic benefits, environmental protection, and strong thixotropic properties. In the second part, regarding the rheological properties (yield stress, stress, and viscosity) associated with the shearing rate, the thixotropy and rheological models were obtained by the indoor tests; the function of the height of the accumulation body and the flow radius was established by an accumulation physical model test, to investigate the actual accumulation process of the thixotropic slurry.

## 2. Materials and Methods

### 2.1. Materials

**Figure 3:**
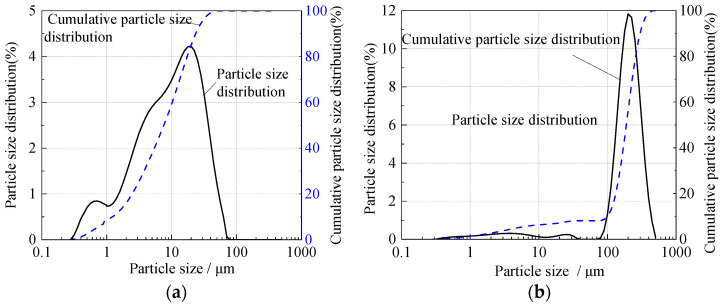
Particle size distribution: (**a**) cement; (**b**) aeolian sand.

Ordinary commercial Portland cement (P.O. 325) and aeolian sand from Yulin, Shaanxi Province, China, were used as the primary components, while a kind of mixed metal hydroxide (MMH) was used as the thixotropic agent. The physical and chemical properties of the materials used in this study are shown in Figure 3 and Table 1. The particle size distribution of cement and the particle size of less than 0.075 mm of the aeolian sand were obtained with a Malvern Mastersizer 2000 Laser Diffraction Particle Size Analyzer (Malvern Instruments Co., LTD; Malvern Worcestershire, United Kingdom), while particle sizes greater than 0.075 mm of aeolian sand were obtained by the sieving method.

### 2.2. Instruments

**Figure 4:**
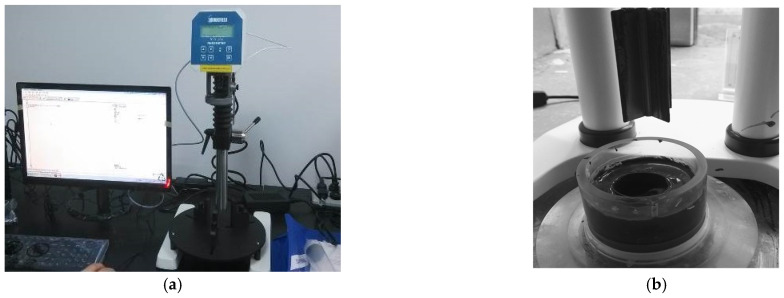
Determination of the slurry rheological parameters using the Brookfield RST−SST: (**a**) rheometer; (**b**) rheological measurement.

The rheological characteristics of the slurry were tested using the Brookfield RST-SST rheometer, as shown in Figure 4. This device can easily realize the shear rate (speed) or shear stress (torque) control. The measuring range of the instrument is in a speed range from 1.66 × 10^−3^ to 21.66 s^−1^ and the viscosity was 0.1 × 10^−3^ to 5.4 × 10^6^ Pa·s, with a torque range of 1 μNm~100 mNm (the corresponding shear stress varies from 0.27 to 69 × 10^3^ Pa, depending on the measurement rotor selection). The rotor used in this measurement is VT-60-30, with a shear stress range of 1.6~1000 Pa.

**Figure 5:**
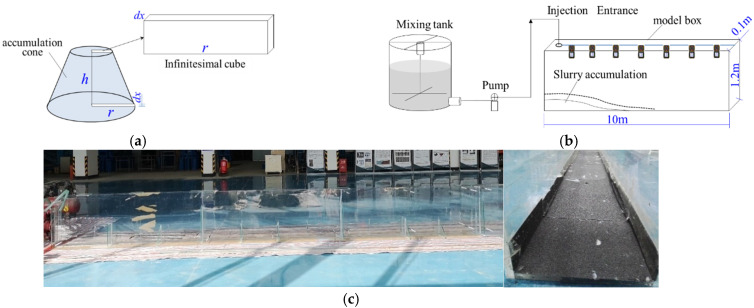
Flow accumulation model test: (**a**) graph for the microelement of the accumulation cone; (**b**) schematic diagram; (**c**) physical view of the flow-stacking model test chamber.

In order to further investigate the actual accumulation process of the developed thixotropic slurry, an accumulation physical model test was performed. The principle of the model is as follows: we assume that the grouting slurry accumulation shape is a truncated cone, and use differential thought, namely, that the circular truncated cone takes a finite length microelement in the axial direction, dx, and the microelement is a rectangular body, as shown in Figure 5.

To prevent the base of the slurry from slipping, the bottom of the box was stuck with coarse sandpaper (Figure 5c). Using a laser rangefinder, the corresponding stacking height under different flow distances was measured with the slurry flow process.

### 2.3. Methods

#### 2.3.1. Sample Preparation

**Figure 6:**
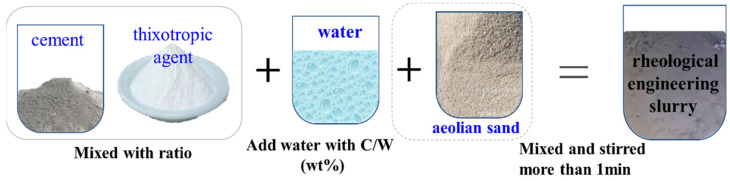
Steps of development of the rheological engineering slurry.

The preparation of the rheological engineering slurry was conducted, based on the following steps illustrated in Figure 6.

**Mix thethixotropic agent into cement**: Firstly, the thixotropic agent should be added to the cement in proportion; we make a well in the mixture in the mixer so that the mixture powder can be dispersed in the water; thus, a strong thixotropic cement slurry will be prepared. Often, many other aggregates, such as aeolian sand, solid waste sand, or tailings sand are mixed in in engineering practice; thus, a strong thixotropic cement mortar will be prepared.

**Rheological engineering slurry preparation**: Water is added in proportion and the mix is stirred for at least 1 min, then the rheological engineering slurry/mortar is prepared.

**Note**: The amount of thixotropic agent and other aggregates, such as aeolian sand, is relative to the cement, and the amount of water is relative to the total weight of solid materials, including the cement, thixotropic agent, and other aggregates. The cement and thixotropic agent were mixed together for 1 min, then water was added, mixing for 1 min to prepare the slurry. The two mixing times were used according to engineering practice.

According to the above preparation steps, a strong thixotropic cement slurry would be prepared when cement was used as the base material if there was no aggregate, otherwise, the strong thixotropic cement mortar would be prepared when the aggregate, such as aeolian sand, solid waste sand, or tailings sand was used as the base material, and the thixotropic agent was used as the auxiliary materials (the amounts of the agent and aggregate both contribute to the ratio of the cement quality). The strongest rheological engineering slurry can be found through tests using different amounts of the thixotropic agent and water. The main rheological properties tests of slurry and mortar (meaning that aggregates, such as aeolian sand, solid waste sand, or tailings sand are used; in this manuscript, the aggregate is aeolian sand) were conducted, as discussed below.

#### 2.3.2. Experimental Design and Procedures

To study the influence of the contents of different components on the properties of strong thixotropic slurry, samples with different mix proportions were designed according to Table 2.

(1) Rheological characterization for a strong thixotropic cement slurry

The water−solid ratio(w/s) of 0.7 was fixed and only the amount of thixotropic agent was changed. The experimental method is: using different standing times, the rotation speed was slowly increased from zero (0 to 1.5 s^−1^ within 15 s), and the static yield stress at 1 s^−1^ was recorded.

(2) Rheological characterization of a strong thixotropic cement mortar

The experiment was based on the assumption that aeolian sand does not participate in the chemical process of the slurry; the amount of aeolian sand (relative to cement) changed only under the conditions of the amount of thixotropic agent compared to cement (8.5%) and the water−solid ratio (w/s) of 0.7 and 0.8.

(3) Flow accumulation test

Using the laser rangefinder, the corresponding stacking height under different flow distances was measured; with the slurry flow process, the accumulation slope curve and the fitting formula could be obtained.

### 2.4. Evaluation Method for Thixotropic Performance

The strong thixotropic performance guarantees the outstanding fluidity of the slurry during infusion/stirring, with good semi-solidity in the standing state, so the mixture could well achieve the filling characteristics of little water precipitation, limited range flow, and steep-angle accumulation because of the special static yield stress, dynamic yield stress, apparent viscosity, and plastic viscosity.

The evaluation of thixotropy is mostly achieved by constructing the relevant indexes of viscosity or the shear force of the slurry system [19]. The thixotropy indexes and test methods often include the thixotropic ring method, the thixotropic index method, etc. [20]. The thixotropic loop method can only measure the thixotropy of slurry in a limited time frame (a period of shear rate increase or decrease). If the rheological parameters continue to develop with the standing time or resting time, the method has some defects and cannot depict the thixotropy character wherein the viscosity or shear force of the slurry increases with standing time. The thixotropic index method reflects the ratio of the apparent viscosity at low speed to the apparent viscosity at high speed, which is the rheological property of the slurry when the equipment is rotating, and that is not a good yardstick of the slurry performance from standing still to stirring. The thixotropy of the strong thixotropic engineering slurry developed in this study is extremely significant over time. Once the slurry starts to flow, the properties of the slurry are completely different from the static state, and the measured thixotropy has been seriously distorted. Therefore, the thixotropic ring method and thixotropic index method cannot fully characterize the strong thixotropy of the rheological parameters with the continuous development of static time.

The main principle of using high thixotropic slurry to fill the goaf areas with steep-angle stacking is that the static yield stress of the slurry is so large that it exceeds the shear stress generated by its own weight and cannot flow; the key rheological parameter of strong thixotropy slurry is static yield stress. Therefore, this study defines the ratio of the difference between the static yield stress, after standing for 10 min and 200 Pa, to the static yield stress of the slurry at the initial time or after re-stirring, to measure the thixotropy of the strong thixotropic slurry. This parameter takes into account both the absolute value of the rheological parameter and the relative value of the rheological parameter before and after the trigger. The value of 200 Pa here represents the comprehensive experimental experience and the previous research experience. The static yield stress of the slurry is about 200Pa, which can basically realize the initial loss of fluidity and become a semi-solid state. Its expression is as follows (1).

(1) $$TI=\frac{\langle \tau_{10}-200\rangle }{\tau_{0}}$$

In the formula, TI is the thixotropic coefficient, $\tau_{0}$ is the static yield stress of the slurry at the initial moment, $\tau_{10}$ is the static yield stress of the slurry after standing for 10 min, and 〈〉 is the Macauley symbol, which means that when $\tau_{10}-200$ is negative, $\langle \tau_{10}-200\rangle$ is 0, and when it is non-negative, it is the function value itself. The static yield stress, $\tau_{0}$ and $\tau_{10},\;$can be measured by the method of shear rate/speed control with the Brookfield RST-SST rheometer; the shear rate is set to increase from 0, then the rotor starts to rotate at the moment when the corresponding shear stress is recorded as the static yield stress (for the convenience of data reading, the static yield stress corresponding to a speed of 1 s^−1^ is taken in the experiment).

## 3. Results

### 3.1. The Optimal Agent Ratio for Thixotropic Cement Slurry

**Figure 7:**
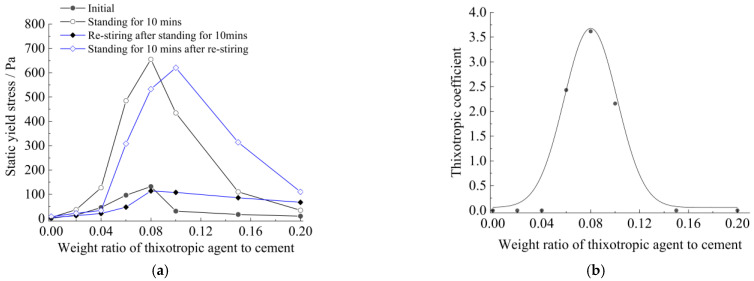
The thixotropy of cement slurry with different thixotropic agents: (**a**) static yield stress−thixotropic agent addition; (**b**) thixotropic coefficient−thixotropic agent addition.

According to the above evaluation method and Formula (1) to calculate thixotropy, the static yield stress and thixotropic coefficient of thixotropic cement slurry, with the amount of thixotropic agent added, are shown in Figure 7, below.

It can be seen that in the gradually increasing process of the thixotropic agent amount (relative to the weight of cement) from 0 to 20%, the static yield stress and the thixotropic coefficient of the slurry first increases and then decreases; they go up to a maximum when the amount of thixotropic agent is between 7% and 10%. The microscopic mechanism of this phenomenon is that the positively charged MMH and negatively charged cement form structures, due to electrostatic attraction. The viscosity of the system increased when the thixotropic agent (MMH) was less than 10%, while the MMH was more than 10%; the structures weakened due to positive electrostatic repulsion.

**Figure 8:**
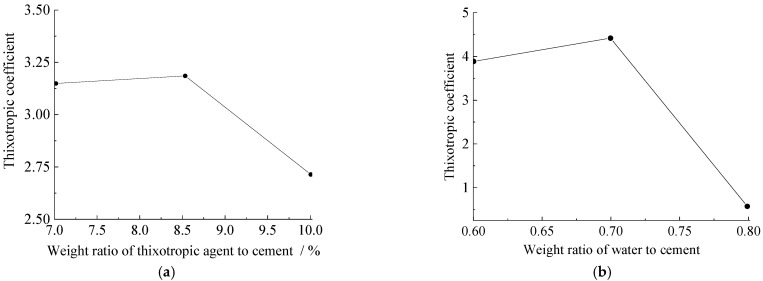
The trend of influence of two factors on the thixotropic coefficient: (**a**) thixotropic agent; (**b**) water−solid ratio.

Based on these results, an orthogonal experiment table was designed (Table 3) to obtain the optimal ratio of the agent, to achieve a strong thixotropic cement slurry (Figure 8).

It was found from the orthogonal experimental results above that 8.5% of the thixotropic agent amount and the 0.7 water−solid ratio were the optimal ratios for thixotropic cement slurry.

### 3.2. The Optimal Agent Ratio for a Strong Thixotropic Cement Mortar

The thixotropic characteristics and the optimal agent ratio for strong thixotropic cement mortar under different amounts of aeolian sand were studied.

The experiment is based on the assumption that aeolian sand does not participate in the chemical process of the slurry, and the amount of aeolian sand (relative to cement) changes only under conditions where the amount of thixotropic agent and cement is fixed.

It was found in the experiments that with an increase in the amount of aeolian sand, the consistency and viscosity of the slurry increased in the initial state. The static yield stresses reached 166.2 Pa and the viscosity was greater than 150 Pa·s when the amount of aeolian sand was added at about 50% of the cement, at a 0.7 water–cement ratio, and the slurry had lost its conventional pumping performance. Therefore, in order to facilitate the normal infusion of the slurry, the static yield stresses should be less than 100 Pa.

**Figure 9:**
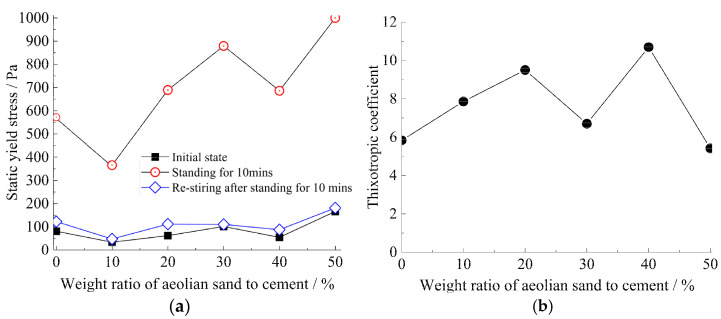
The thixotropy of cement mortar (at a water–solid ratio = 0.7): (**a**) relationship between yield stress and aeolian sand amount; (**b**) thixotropic coefficient−aeolian sand amount.

**Figure 10:**
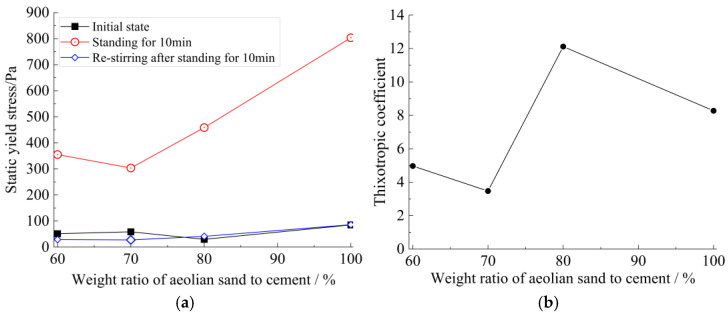
The thixotropy of cement mortar (at a water–solid ratio = 0.8): (**a**) relationship between yield stress and aeolian sand addition; (**b**) thixotropic coefficient−aeolian sand addition.

The rheological curves of strong thixotropic cement mortar under different aeolian sand dosages, under the optimal thixotropic agent content, are shown in Figure 9 and Figure 10 below.

As depicted in Figure 9 and Figure 10, the thixotropy increases first and then decreases with the aeolian sand addition. The main reason is that the amount of aeolian sand causes the initial rheology of the slurry to deteriorate, resulting in a larger initial slurry consistency and a yield stress close to or even greater than 200 P, and it nearly loses fluidity. It is necessary to increase the water–solid ratio from 0.7 to 0.8 when the amount of aeolian sand is more than 50%.

It was observed that the optimal ratios are w/s = 0.7 and the weight ratio of aeolian sand to cement ≤ 50%, or w/s = 0.8, and the weight ratio of aeolian sand to cement > 50% for a strong thixotropic cement mortar.

### 3.3. The Rheological Properties of Slurry and Mortar

**Figure 11:**
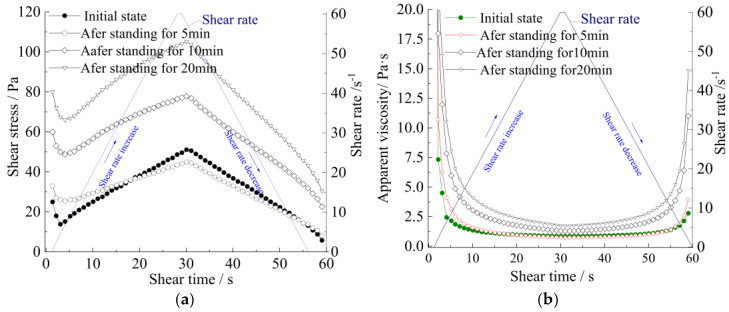

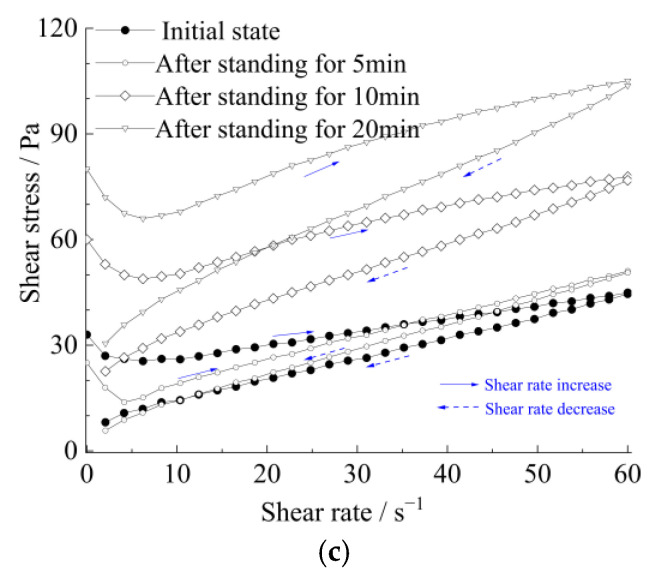
Rheological curves of strong thixotropic cement slurry (the proportion of thixotropic agent = 8.5%, water−solid ratio = 0.7): (**a**) shear stress−shear time; (**b**) apparent viscosity−shear time; (**c**) shear stress−shear rate.

**Figure 12:**
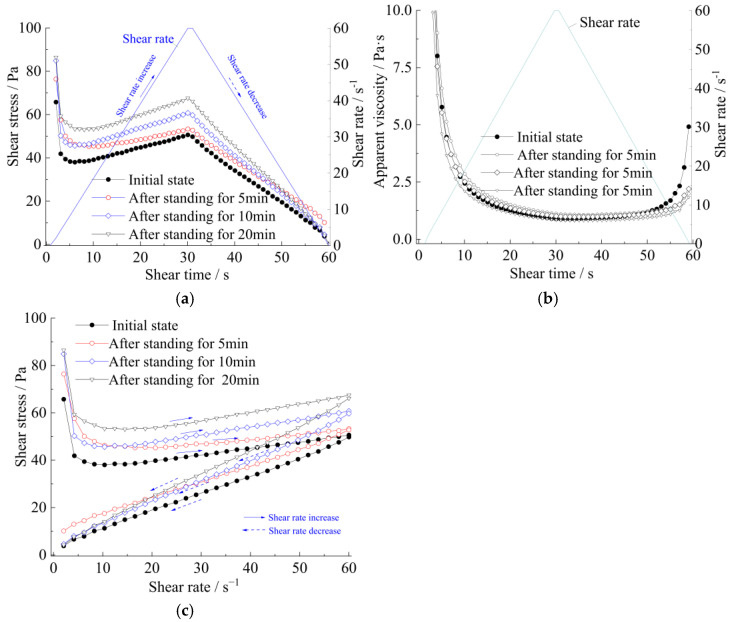
The thixotropy of cement mortar (at a water−solid ratio = 0.8): (**a**) shear stress−shear time; (**b**) apparent viscosity−shear time; (**c**) shear stress−shear rate.

**Figure 13:**
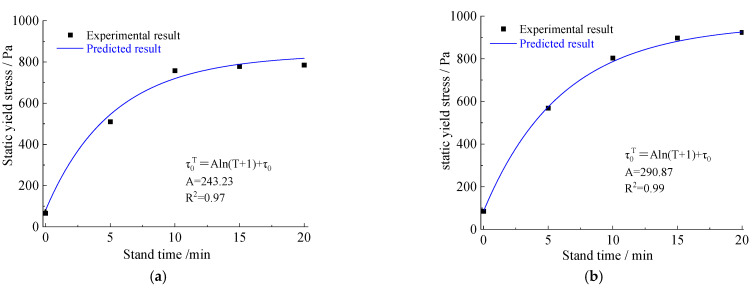
Curve of the static yield stress of strong thixotropic slurry with standing time: (**a**) strong thixotropic cement slurry (the proportion of thixotropic agent to cement = 8.5%, water–solid ratio = 0.7); (**b**) strong thixotropic cement mortar (the proportion of thixotropic agent to cement = 8.5%, water−solid ratio = 0.8, the proportion of aeolian sand to cement = 100%).

Figure 11 and Figure 12 show the relationships between shearing stress and shearing time or shearing rate, while Figure 13 shows the relationships between yield stress and resting time. The test results demonstrate that the yield stress at rest does not increase as a linear progression [17].

Both results show that the yield stress increases with the resting time, which is consistent with Equation (2):

(2) $$\tau_{0}^{T}=f(\tau_{0},T)=A\ln(T+1)+\tau_{0}$$

where $\tau_{0}^{T}$ is the static yield stress (Pa) of the slurry after different standing times T(s), $\tau_{0}^{}$ is the static yield stress of initial mixing of the slurry, and A is the dimensionless fitted parameters.

As expected, after different resting times, both the yield stress $\tau_{0}^{T}$ of thixotropic cement slurry and the mortar increased with increasing T. $\tau_{0}^{T}=\tau_{0},\;$when T = 0. A natural logarithmic function was used to fit the relationships between yield stress and rest time.

Meanwhile, the curves of apparent viscosity with the shear rate and shear stress, along with the shear rate, show that the rheological model of the strong thixotropic slurry is extremely related to both shear rate and standing time; the rheological character is caused by the imbalance between the internal structure and the recovery of the slurry system. In Figure 11 and Figure 12, it can be seen that the stress decays sharply with shearing (the shear rate is more or less than 10 s^−1^), then the stress increases with the increase in shear rate (the shear rate is more than 10 s^−1^). The stress increase in the slurry is greater than in the mortar.

Therefore, through these experimental results, combined with theoretical analysis, the following rheological model can be obtained:

(3) $$\tau =\tau_{0}^{T}\left(1-e^{-m\frac{\tau_{0}}{\tau_{0}^{T}}\dot{\gamma }}\right)+\tau_{0}^{T}a^{\eta_{p}\dot{\gamma }}$$

(4) $$\eta =\frac{\tau }{\dot{\gamma }+1}$$

where τ is the shear stress of the slurry (Pa), η is the apparent viscosity (Pa·s), $\eta_{p}$ is the plastic viscosity (Pa·s), $\tau_{0}^{T}$ is the static yield stress (Pa) of the slurry after different standing times T (s), $\mu_{0}^{T}$ is the yield viscosity(Pa·s) of the slurry at different standing times T, and $\tau_{0}^{}$ is the static yield stress of initial mixing of the slurry, $\mu_{0}^{}$ is the yield viscosity of initial mixing of the slurry, and $\dot{\gamma }$ is the shear rate. m,a,A are the dimensionless fitted parameters.

Formula (3) is divided into two parts. $\tau_{0}^{T}\left(1-e^{-m\frac{\tau_{0}}{\tau_{0}^{T}}\dot{\gamma }}\right)$ represents the restoration of the structure in the thixotropic slurry system, and the second part $\tau_{0}^{T}a^{\eta_{p}\dot{\gamma }}$ represents the loss of the resistance to shear stress caused by the shear disturbance in the slurry system.

**Figure 14:**
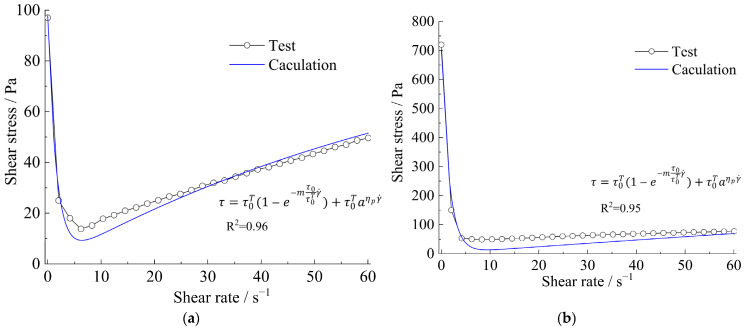
The relationship between shear stress and the shear rate of strong thixotropic cement slurry: (**a**) initial time (**b**) after standing for 10 min.

**Figure 15:**
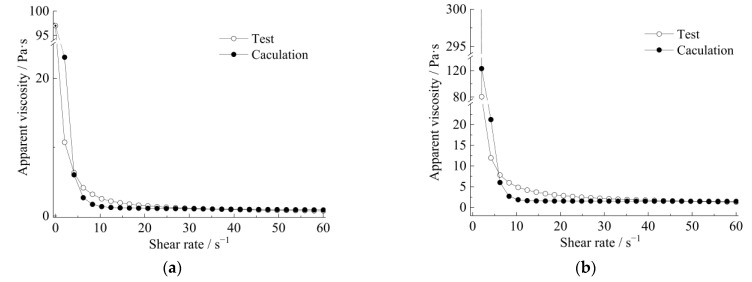
The relationship between apparent viscosity and the shear rate of a strong thixotropic cement slurry: (**a**) initial time (**b**) after standing for 10 min.

**Figure 16:**
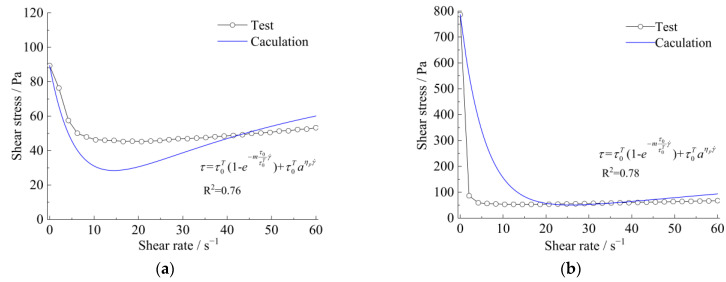
The relationship between shear stress and the shear rate of a strong thixotropic cement mortar: (**a**) initial time (**b**) after standing for 10 min.

**Figure 17:**
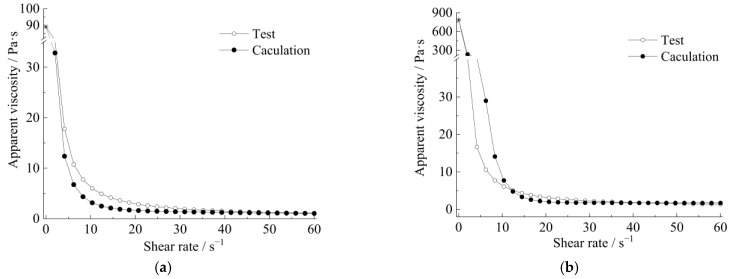
The relationship between apparent viscosity and the shear rate of a strong thixotropic cement mortar: (**a**) initial time (**b**) after standing for 10 min.

The rheological characteristics of cement slurry and mortar at the initial time and after standing for 10 min are shown in Figure 14, Figure 15, Figure 16 and Figure 17. The fitted parameters are shown in Table 4. The equation was validated using the experimental results.

Figure 14 and Figure 16 show that after 10 min, the rapid buildup of yield stress resulted in the formation of a semi-stagnant semi-solidified state, and the cement mortar is more pronounced. When the slurry or cement mortar was re-mixed, the structures were easily broken, while the stress decayed and exhibited shear-thinning behavior.

In summary, the rheological model of a strong thixotropic slurry or mortar was established, and the calculated values of the established rheological model and the experimental values have an excellent relationship.

The possible existence of a buildup of yield stress in strong thixotropic engineering slurry may be attributed to the fact that the complex particulate structural networks are rebuilt. The particulate network structures are easily broken, which results in stress decay by shearing.

### 3.4. Flow Accumulation Properties

**Figure 18:**
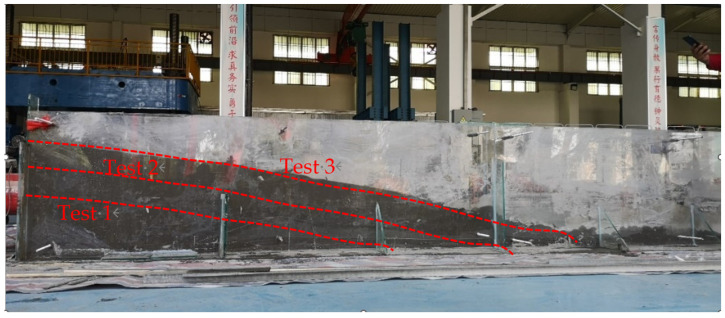
Diagram of the model test for the flow-packing of a strong thixotropic cement mortar.

The filling and accumulation process of the developed strong thixotropic slurry is obtained, as shown in Figure 18. Tests 1, 2, and 3 show that three measurements were taken during the flow and accumulation process of the slurry.

Based on the experimental measurements recorded, the flow curvature h=h(x) is obtained, as shown in Formula (5). The formula can be used to estimate the maximum flow range needed to fill a certain height space:

(5) $$h=h_{0}+B(e^{-x/C}-1)$$

where h is the height of the accumulation body (m) along the slurry flow radius x (m), $h_{0}$ is the height of the goaf (m), x is the slurry flow radius (m), and B and *C* are the dimensionless fitted parameters, which were fitted as in Table 5.

**Figure 19:**
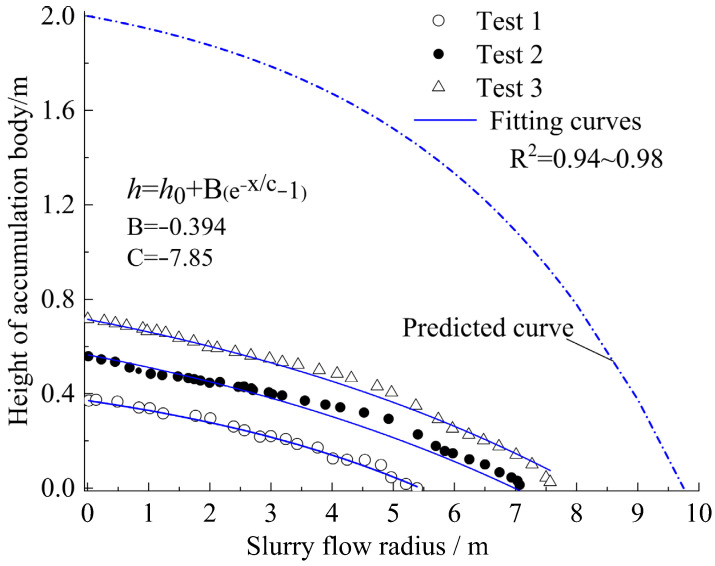
Slope lines of the flow accumulation.

The thixotropic behavior of the slurry is clearly reflected in Figure 19 because of its high accumulation while there is a short range of flow.

According to the formula and Figure 19, it is estimated that the maximum flow radius is 9.48 m, under the 1.8 m high of the goaf.

## 4. Conclusions

In this study, the optimal agent ratio for thixotropic cement slurry/mortar is obtained by indoor tests; the rheological agent should be 7.5% of the weight of the cement, the water–solid ratio is in the range of 0.7~0.8, and the aeolian sand should be 0~1.5 times that of the cement.

The particular rheological properties of slurry and mortar are obtained using a Brookfield RST-SST rheometer. The results showed that aeolian sand can be used as a thixotropic cement mortar for a backfilling treatment for underground voids (mine goaf), whereby the static yield stress increases non-linearly, compared to the existing thixotropic models [21,22]. The stress decays sharply with shearing (the shear rate is more or less than 10 s^−1^), then the stress increases with the increase in shear rate (the shear rate is more than 10 s^−1^).

Then, a natural logarithmic function between yield stress and rest time (only one parameter is required and is easily determined by fitting the experimental data in different resting experiments), an exponential function with two parts for the stress–shear rate (in the rheology model, only 3 parameters are required and are easily determined by fitting the experimental data in the stress decay experiments), and an exponential function for the accumulation law (only 2 parameters are required and are easily determined by fitting the experimental data in the accumulation experiment) are obtained.

## Figures and Tables

**Table 1:** The thixotropic agent properties.

Items	Index
Appearance	White powder
Electric potential/mV	≥35
Sieve residue (aperture 0.25 mm)	≤5
Acid solubility %	≥95

**Table 2:** Samples of different mixture proportions.

Mix No.	Cement/g	Thixotropic Agent/g	Aeolian Sand/g	Water/g	Note
S1	300	0	0	210	thixotropic cement slurry
S2	300	6	0	214.2	
S3	300	12	0	218.4	
S4	300	18	0	222.6	
S5	300	24	0	226.8	
S6	300	30	0	231	
S7	300	45	0	241.5	
S8	300	60	0	252	
m1	300	25.5	30	248.85	thixotropic cement mortar under w/s = 0.7
m2	300	25.5	60	269.85	
m3	300	25.5	90	290.85	
m4	300	25.5	120	311.85	
m5	300	25.5	150	332.85	
m6	300	25.5	180	404.4	thixotropic cement mortar under w/s = 0.8
m7	300	25.5	210	428.4	
m8	300	25.5	240	452.4	
m9	300	25.5	270	476.4	
m10	300	25.5	300	500.4	

**Table 3:** Orthogonal experiment table.

Level	1	2	3
Factor			
Thixotropic agent amount/%	7	8.5	10
Water−solid ratio	0.6	0.7	0.8

**Table 4:** Fitting parameters of the rheological curve of a strong thixotropic slurry.

Parameters		Plastic Viscosity $\eta_{p}/\mathrm{Pa}\cdot s$	Static Yield Stress at the Time of Initial Mixing $\tau_{0}^{}/\mathrm{Pa}$	*A*	*m*	*a*	Yield Stress Pa after Standing for 10 Min $\tau_{0}^{10}/\mathrm{Pa}$
Value	slurry	0.586	97.76	251.62	0.0125	0.3441	701.11
	Mortar	0.672	89.38	283.61	0.0176	0.873	786.86

**Table 5:** Fitting parameters of flow accumulation.

Parameter	h_0/_m	B	C
Test			
1	0.37	−0.394	−7.85
2	0.56		
3	0.71		

## Data Availability

The data used to support the findings of this study are available from the corresponding author or first author upon request.
